# Thermodynamic Modeling and Analysis of an Optical Electric-Field Sensor

**DOI:** 10.3390/s150407125

**Published:** 2015-03-24

**Authors:** Xia Xiao, Yan Xu, Zexing Dong

**Affiliations:** State Key Laboratory of Advanced Electromagnetic Engineering and Technology, Huazhong University of Science and Technology, 1037 Luoyu Road, Wuhan 430074, China; E-Mails: xuyan919@hust.edu.cn (Y.X.); dzexing@163.com (Z.D.)

**Keywords:** optical electric-field sensor, thermodynamic model, sensor crystal, optical properties, stability

## Abstract

The stability of the optical electric field sensor (OEFS) in actual operation is affected by environmental factors such as temperature and SF_6_ (sulfur hexafluoride). To analyze the operational environment parameters affecting the optical properties of crystals, a thermodynamic model of the OEFS in which the optical properties of the crystal are changed by the first-order effects and the second-order effects was established. The intensity parameters such as electric, stress and temperature fields were introduced. The theoretical analysis results show that under temperature, stress and electric field conditions, the optical properties of the sensing crystals are no longer changed only by the electro-optic effect, but also by the temperature and the stress fields. Further synthesis suggests the expected optical property changes under the effect of the environment fields. OEFS tests show that the accuracy of OEFS is dependent on temperature with a ratio error of −0.8%~1.5% in the temperature range from −25 °C to +40 °C.

## 1. Introduction

Passive electronic voltage transformers (PEVTs) based on the principle of the electro-optic effect have bright prospects in high voltage grade applications because of a series of advantages they display such as their wide measuring range, good frequency characteristics, a simple insulation structure and higher safety [1,2,3].

However, at present PEVTs can’t be widely applied in power systems, mainly because the long term running stability of the optical electric field sensor (OEFS) cannot meet the power grid requirements. There have been many studies aimed at improving the stability of OEFS, such as employing software compensation or using a double optical path structure to improve the temperature performance of OEFS [4,5,6,7,8,9].

The temperature effect on OEFS shows that macroscopically there is a certain relationship between the sensor output and the temperature. In [9], Filippov identified in the experiments the temperature characteristics of each OEFS in actual operation, and obtained the operational temperature of the sensor. Thus the dependence of OEFS on temperature could be compensated to some extent. In the 1990s, Lee improved the temperature stability of OEFS from ±7.0% to ±0.75% within −2 °C~65 °C with dual light path compensation [10]. In recent literature, the accuracy of OEFS was reported to be improved to ±0.5% within −40 °C~+60 °C by introducing a reference voltage for comparison [11].

**Figure 1:**
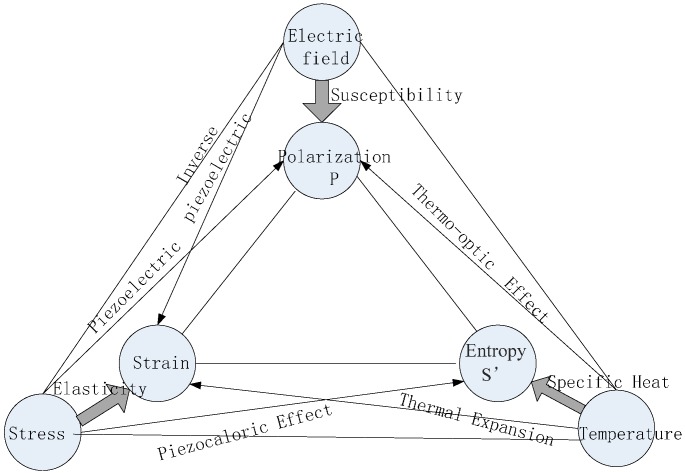
The relationship between various parameters of a crystal system.

In actual operation the electro-optic sensing crystal of the OEFS interacts not only with the electric field to be tested, but also with the temperature field and stress field. The electric, temperature and stress fields can change the optical properties of electro-optic crystals. The relationships between the various parameters of the crystal system are shown in Figure 1. Therefore, in any further study of the stability of PEVT, the sensor must be seen as a system that can respond in many ways to a series of environmental fields.

## 2. The Principle and Scheme of OEFS

**Figure 2:**
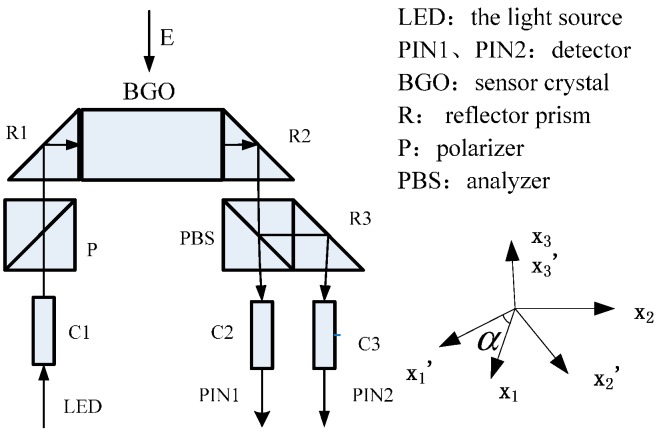
The principle of OEFS.

OEFS based on bismuth germinate (BGO) crystals are a common research subject [12,13], and their basic principle of operations is known as the linear electro-optic effect. The transverse modulation OEFS principle diagram is shown in Figure 2. The crystal refractive index changes with the electric field *E*, which is called the linear electro-optic effect. The variable $\Delta \beta =\beta -\beta_{0}$ of the reverse dielectric tensor β($\beta =\frac{1}{{n_{0}}^{2}}$) is usually used to describe the changes of the optical properties of crystals, where, *β*_0_ and *β* are the reverse dielectric tensor with electric field and without electric field, respectively, and *n*_0_ is the refractive index.

Without an electric field, the optical properties of a BGO crystal could be expressed with the refractive index ellipsoid equation as follows [14]:

(1) $$\beta_{0}x_{1}^{2}+\beta_{0}x_{2}^{2}+\beta_{0}x_{3}^{2}=1$$

With an electric field, the inverse dielectric tensor β would be changed as:

(2) $$\Delta \beta_{ij}=\gamma_{ijk}E$$

In Equation (2), *E* is the electric field, $\gamma_{ijk}$ is the linear electro-optic coefficient matrix, and:

$$\gamma_{ijk}=\left[\begin{array}{ccc} 0 & 0 & 0\\ 0 & 0 & 0\\ 0 & 0 & 0\\ \gamma_{41} & 0 & 0\\ 0 & \gamma_{41} & 0\\ 0 & 0 & \gamma_{41} \end{array}\right]$$

In the ideal case, when the light direction is along the $\bar{1}10$ direction of the BGO crystal and the electric field $E_{k}$ is applied perpendicular to the 001 surface of the crystal, the birefringence phase retardation caused by electro-optic effect is:

(3) $$\delta =\frac{\pi l}{\lambda }{n_{0}}^{3}\gamma_{41}E_{k}$$

where λ is the wave length of input light, $\gamma_{41}$ is the electro-optic coefficient of the crystal, *l* is the length of the light path through the crystal. From Equation (3), the corresponding applied electric field can be obtained by measuring the birefringence phase retardation.

**Figure 3:**
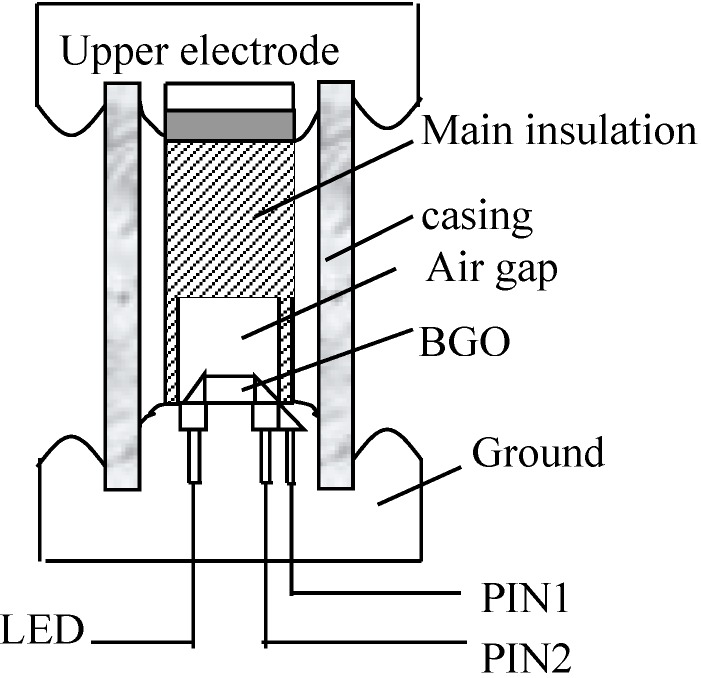
Structural diagram of a sensor.

In OEFS based on the transverse modulation structure, the term $E_{k}$ in Equation (3) is the electric field where the light passes through the crystal, which is generated by the appropriate electrode structure. As shown in Figure 3, a measured voltage is applied between the upper electrode and the ground electrode, the middle of which is supported by the casing. The main insulation, sensing crystals and the air gap make up the medium to produce the electric field $E_{k}$ to be tested. To ensure a sufficient insulation intensity, the sensor is placed in a sulfur hexafluoride (SF_6_) gas environment.

## 3. Thermodynamic Modeling of OEFS

The physical properties of crystals describe the relationship between the independent variable and the dependent variable. The physical basis of OEFS to measure the electric field is the linear electro-optic effect, that is, the change in the optical properties of the sensing crystal with an electric field. However, in practical cases, what affects the crystal optical properties is not only the electric field, but also the temperature field and stress field.

OEFS operates under a gas pressure and temperature environment, and OEFS works with the surrounding environment under a thermal equilibrium state. The relationship between the environmental parameters and the physical properties of the crystal indicated in the thermodynamic model is shown in Figure 1. The intensity parameters such as temperature *θ*, stress *T* and electric field intensity E show an effect on the crystal; extensive parameters such as the entropy $S^{´}$, the electric polarization P and strain *S* show the dependent variables caused by the effect of the crystal. The BGO crystals used for the OEFS sensor belong to the cubic crystal system with no pyroelectric effect.

In Figure 1, the effects marked with a thick arrow include the main effects, which describe the relationship between the same kind of intensity parameters and the extensive parameters, such as thermo main effect, electrical primary effect and mechanical effect. The relationship between the independent variables and other extensive parameters marked with a thin arrow are the cross effects, such as mechanical effect, electrothermo effect and thermoelastic effect.

As discussed above, various physical properties of the crystal are intrinsically linked. Under different conditions, the same physical properties tend to be different. Therefore, in the study of the crystal properties, all kinds of possible effects must be taken into consideration, in particular, the thermodynamic relationship between the various processes.

The present study is focused on dielectric polarization, because the optical properties of the crystal and its polarization or dielectric properties have a direct relationship. In Figure 1, the intensity parameters marked by the arrow pointing directly to the crystal electric polarization are the electric field, stress field and temperature field. The relationship among them are first-order effects including the electro-optic effect, elastic-optic effect and thermo-optic effect. There are still some second-order effects. One is the electrostrictive effect generated by the electric field, in which the inverse piezoelectric effect and electrostriction strain caused by the electric field produce a strain, while the elastic-optic effect by the strain changes the crystal polarizability; the other is the photoelastic effect induced by the stress with the change of temperature.

Therefore, the analysis of crystal optical properties in the thermodynamics system should be done taking into consideration the interactions of electric field, temperature field and stress field. The electric field causes the first-order electro-optic effect. The temperature field on the one hand causes the thermo-optic effect, and produces the elastic-optic effect created by the unequal heat within the crystal or temperature mutation as the effect of stress would. Gas pressure would also cause elastic-optic effects in the crystal.

Changes of susceptibility state can be expressed with differential relationship between the dependent variable and independent variable as:

(4) $$dP_{i}={\left(\frac{\partial P_{i}}{\partial T_{kl}}\right)}_{E,\theta }dT_{kl}+{\left(\frac{\partial P_{i}}{\partial E_{j}}\right)}_{T,\theta }dE_{j}+{\left(\frac{\partial P_{i}}{\partial \theta }\right)}_{T,E}d\theta$$

In Equation (4), the right first item is for the piezoelectric modulus, the second item is for the electric susceptibility and the third item is for the pyroelectric coefficient. Integrating the intensity parameter, the function of the crystal polarization intensity as the intensity parameters for a certain value is:

(5) $$P_{i}=p_{ijkl}^{\theta }T_{kl}+\varepsilon_{0}\chi_{ij}^{T,\theta }E_{j}+p_{i}^{T}\Delta \theta$$

where $\chi_{ij}^{T,\theta }$ is the crystal polarizability. $p_{ijkl}^{\theta }$ is the elastic optic effect coefficient. $p_{i}^{T}$ is the pyroelectric coefficient. From Equation (5), under the effects of temperature, stress and electric field on the OEFS, the sensing crystal optical properties change not only with the electro-optic effect, but also with the temperature field and the stress field.

## 4. Analysis of OEFS Optical Properties in the Thermodynamic System

### 4.1. Effect of Temperature on the Optical Properties of Crystals

From Figure 1, the effect of temperature on the optical properties of BGO includes two parts. One is the polarization caused by the thermo-optic effect, and the other is the polarization caused by the elastic-optic effect.

#### 4.1.1. The Thermo-Optic Effect on BGO Crystals

BGO crystals belong to the cubic system, in which the thermo-optic effect is expressed as [14]:

(6) Δβ=bΔθ

In Equation (6), Δθ is the temperature variable, b is the thermo-optic effect coefficient. Supposing that the coordinate axis is the spindle of the refractive index ellipsoid, the thermo-optic coefficient matrix is:

$$b_{ij}=\left[\begin{array}{ccc} b_{11} & 0 & 0\\ 0 & b_{11} & 0\\ 0 & 0 & b_{11} \end{array}\right]$$

#### 4.1.2. The Elastic-Optic Effect Caused by Thermo Stress in BGO Crystals

When the environmental temperature changes in a crystal, the optical properties of BGO would change with the thermo stress due to the elastic-optic effect. The elastic-optic effect can be expressed as:

(7) $$\Delta \beta =P_{ijkl}{\vec{T}}_{kl}(i,j,k,l=1,2,3)$$

In Equation (7), $P_{ijkl}$ is the elastic-optic coefficient. Equation (7) could be represented with components as:

$$\Delta \beta_{ij}=P_{ijkl}{\vec{T}}_{kl}$$

where $P_{ijkl}$ is a four order tensor. $\Delta \beta_{ij}$ is a symmetric tensor. ${\vec{T}}_{kl}$ is the symmetric tensor too, so both of the first two subscripts i,j and the latter two subscripts k,l have a symmetric permutation. The elastic-optic coefficient matrix $P_{ijkl}$ could be simplified as [14]:

$$\left[\begin{array}{cccccc} p_{11} & p_{12} & p_{12} & \; & \; & \;\\ p_{12} & p_{11} & p_{12} & \; & \; & \;\\ p_{12} & p_{12} & p_{11} & \; & \; & \;\\ \; & \; & \; & p_{44} & & \\ \; & \; & \; & \; & p_{44} & \\ \; & \; & \; & \; & \; & p_{44} \end{array}\right]$$

As shown in Figure 2, the crystal together with two triangular prisms is bonded on the ground electrode. When the environmental temperature changes, the BGO crystal will be deformed because of heat expansion and cold contraction. In the coordinate system as shown in Figure 2, the BGO crystal could expand freely because of the lack of constraints in the direction parallel to the electric field, while the deformation in the light passing direction $X_{2}^{´}$ will be subject to constraints because the crystal and the triangular prism have different expansion coefficients. According to the thermal stress theory, thermal stress will be produced. Selecting the coordinate system $X_{1}$, $X_{2}$, $X_{3}$ as the principle stress axes, the shear stress component is zero. $T_{1}$, $T_{2}$ and $T_{3}$ are the main stresses parallel to $X_{1}$, $X_{2}$ and $X_{3}$ respectively. $T_{4}$, $T_{5}$ and $T_{6}$ are the shear stresses in plane $X_{1}X_{2}$, $X_{2}X_{3}$ and $X_{3}X_{1}$ respectively. Because of the smaller size of BGO crystals and the slower temperature change, the temperature gradient can be regarded as zero. Then:

$$T_{1}=T,\;T_{2}=-T,\;T_{3}=T_{4}=T_{5}=T_{6}=0$$

### 4.2. The Elastic Optic Effect Caused by SF_6_ Pressure

OVS operates under a SF_6_ environment and the optical properties of BGO crystals are changed by photoelastic effect due to compressive stress of SF_6_. When SF_6_ is in equilibrium, in the coordinate system as shown in Figure 2, $P_{1}$, $P_{2}$ and $P_{3}$ are the main stresses parallel to $X_{1}$, $X_{2}$ and $X_{3}$ respectively. $P_{4}$, $P_{5}$ and $P_{6}$ are the shear stresses in plane $X_{1}X_{2}$, $X_{2}X_{3}$ and $X_{3}X_{1}$, respectively. The compressive stress is:

$$P_{1}=P_{2}=P_{3}=-P,\;P_{4}=P_{5}=P_{6}=0$$

### 4.3. Effect of All Environment Fields on the Optical Properties of Crystals

In the sensing system shown in Figure 1, an electric field is applied perpendicular to the 001 direction of the crystal, then *E*_1_ = *E*_2_ = 0, *E*_3_ = *E*, and the light pass through the crystal along the $\bar{1}10$ direction. With the effect of thermo stress caused by the temperature variation Δθ, and with the effect of SF_6_ gas compressive stress, the change of crystal optical properties is synthetical for each effect. That is:

(8) $$\Delta \beta =\gamma_{ijk}E_{k}+b\Delta \theta +p_{mn}T_{n}+p_{mn}P_{n}$$

Substituting the matrix $\gamma_{ijk}$, b and $p_{mn}$ into Equation (8), then:

(9) $$\Delta \beta =\left[\begin{array}{c} (p_{11}-p_{12})T-(p_{11}+2p_{12})P+b_{11}\Delta \theta \\ -(p_{11}-p_{12})T-(p_{11}+2p_{12})P+b_{11}\Delta \theta \\ -(p_{11}+2p_{12})P+b_{11}\Delta \theta \\ 0\\ 0\\ \gamma_{41}E \end{array}\right]=\left[\begin{array}{c} A\\ B\\ C\\ 0\\ 0\\ \gamma_{41}E \end{array}\right]$$

Combining Equation (1), the refractive index ellipsoid equation indicating the optical properties of crystal is:

(10) $$\begin{array}{l} (\beta_{0}+A+2\gamma_{41}E\sin2\alpha ){x_{1}^{´}}^{2}-(\beta_{0}+B-2\gamma_{41}E\cos2\alpha ){x_{2}^{´}}^{2}+(\beta_{0}+C){x_{3}^{´}}^{2}\\ +[(-A+B)\sin2\alpha +\gamma_{41}E\cos2\alpha ]x_{1}´x_{2}´=1 \end{array}$$

where $x_{1}´$, $x_{2}´$, $x_{3}´$ is the new coordinate system of the refractive index ellipsoid. The coefficient of the item $x_{1}´x_{2}´$ should be zero, namely:

(11) $$(-A+B)\sin2\alpha +\gamma_{41}E\cos2\alpha =0$$

and so:

(12) $$\tan2\alpha =\frac{2\gamma_{41}E}{B-A}=\frac{\gamma_{41}E}{(p_{11}-p_{12})T}$$

α is the rotation angle that the new index ellipsoid coordinate system rotates around the axis $x_{3}$, and is dependent on the applied electric field and temperature. α=±π/4 is the ideal rotation angle without stress.

From Equation (12), under the combination of environmental fields, the refractive index ellipsoid principle axis of the crystal is rotated around the $x_{3}$ axis with a rotation angle α, and the change of the three main shafts length is not equal. The birefringence phase retardation due to environmental fields is:

(13) $$\Delta \varphi =\frac{2\pi }{\lambda }{n_{0}}^{3}l[\gamma_{41}E\sin2\alpha +(p_{11}-p_{12})T]$$

For the summation terms in the bracket in Equation (13), the former is the phase retardation caused by the electric field, whereas the latter is the phase retardation caused by thermal stress due to the change of temperature. The phase retardation caused by the thermo-optic effect is zero because of the optical symmetry of BGO crystals.

## 5. Tests of OEFS

**Figure 4:**
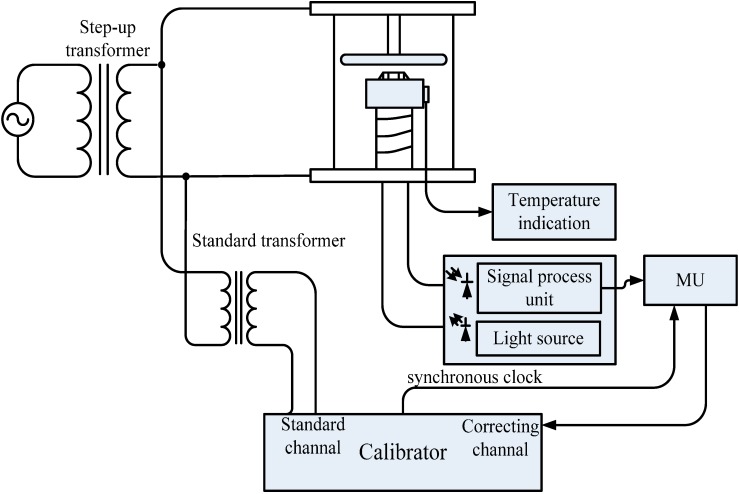
OEFS Tests Schematic Diagram.

The OEFS is fixed inside the insulation sleeve as a test device which is filled with SF_6_ gas. The test principle diagram of the sensor system is shown in Figure 4. The step-up transformer outputs the measured voltage. The voltage is connected to the high voltage electrode of the test device through a wire, making the electric field distribution inside the BGO crystal approximately equal to the actual runtime distribution. The light output of the OEFS is converted to an electrical signal which is amplified and then transferred to the merging unit (MU) where the output of MU is connected to the calibrated channel of the calibrator. In addition, the voltage to be measured is transformed by the standard voltage transformer to small voltage signals which are connected to the standard channel of the calibrator. The ratio error and the phase error of OEFS are calculated in the calibrator.

**Figure 5:**
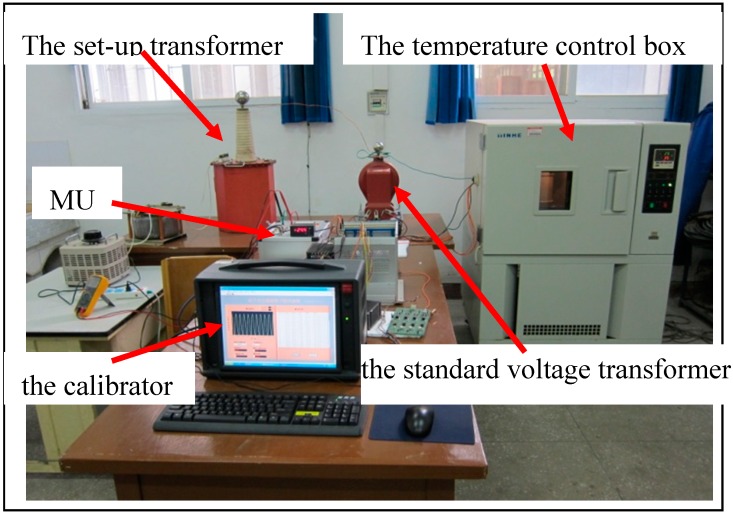
The Temperature Test Photo of OEFS.

**Figure 6:**
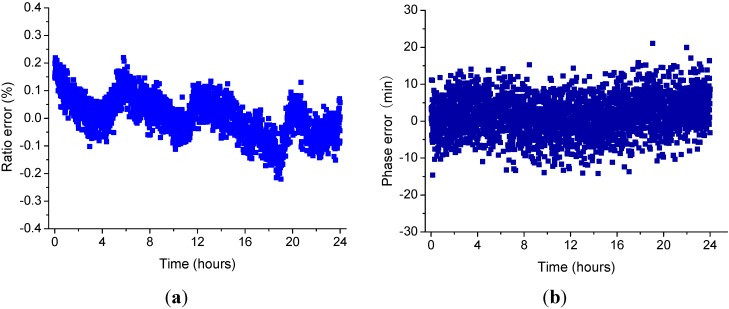
(**a**) The Ratio Error Curve within 24 h; (**b**) The Phase Error Curve within 24 h.

**Figure 7:**
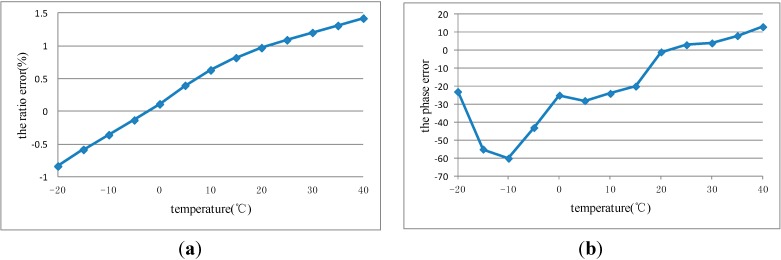
(**a**) The Ratio Error Curve with Temperature; (**b**) The Phase Error Curve with Temperature.

The insulating sleeve is placed in the temperature control box. The measured voltage is applied to the high-voltage electrode. A photograph of the test system is shown in Figure 5. The temperature characteristics of OEFS were obtained. Figure 6 shows the ratio error curve and the phase error curve of OEFS at room temperature within 24 h at the applied voltage up to 10 kV. Figure 7 shows the ratio error curve and the phase error curve of OEFS with temperature at an applied voltage up to 10 kV. From Figure 6, the accuracy of OEFS within 24 h at room temperature is within ±0.2%. When the range of temperature is much smaller and with slow changes, the stress produced in the crystal will be less. From Equation (13), the second term with the stress T will be less, so the accuracy of the OEFS can be kept within ±0.2%. However, when the temperature changes greatly and the rate of change is larger, the thermal stress produced in the crystal will change with temperature and the accuracy of OEFS will be worse. From Figure 7, the ratio error of OEFS is within −0.8%~1.5%, and the phase error is within −60’~+15’ with the temperature range from −20 °C to +40 °C.

## 6. Conclusions

The nature of OEFS is that an external electric field changes their optical properties. The analysis of a thermodynamic system model of OEFS in actual operation suggests that the external temperature field and pressure field will also change the optical properties of BGO crystals, thus resulting in instability of OEFS. The tests results show that the accuracy of OEFS at room temperature is within ±0.2%, but dependent on temperature.
